# Anticancer activity of MDM2 inhibition in 2D and bioprinted 3D retinoblastoma cell models

**DOI:** 10.3389/fphar.2025.1692250

**Published:** 2025-11-06

**Authors:** Francesca Bompan, Giada Lodi, Rebecca Foschi, Anna Dipinto, Lucia Carmela Cosenza, Fabio Casciano, Paolo Severi, Anna Sanvido, Lorenzo Caruso, Luisa Giari, Giorgio Zauli, Rebecca Voltan, Arianna Romani

**Affiliations:** 1 Department of Environmental and Prevention Sciences, University of Ferrara, Ferrara, Italy; 2 Department of Translational Medicine and LTTA Centre, University of Ferrara, Ferrara, Italy; 3 Department of Environmental and Prevention Sciences and LTTA Centre, University of Ferrara, Ferrara, Italy; 4 Department of Translational Medicine, University of Ferrara, Ferrara, Italy; 5 Research Department, King Khaled Eye Specialistic Hospital, Riyadh, Saudi Arabia

**Keywords:** retinoblastoma, nutlin-3a, Y79, Weri-Rb1, bioprinted model

## Abstract

Retinoblastoma is the most common childhood tumor affecting the retina. Pharmacological resistance or delayed intervention leads to the loss of vision. Therefore, novel therapeutic strategies need to be assessed in preclinical models that mimic the *in vivo* tumor. This project aims to investigate the anticancer activity of the MDM2 inhibitor, nutlin-3a, in the treatment of retinoblastoma using both conventional 2D *in vitro* models and more-realistic 3D-bioprinted models. Unlike many cancers, retinoblastoma presents a p53 wild-type phenotype, making the p53 pathway a promising target for pharmacological treatment via MDM2 inhibitors. Initially, nutlin-3a was tested on Y79 and Weri-Rb1 retinoblastoma 2D cell line cultures. A significant, concentration-dependent reduction in cell viability was observed as early as 24 h, associated with cell cycle blockade in both S and G2/M phases, assessed through cytofluorimetric analysis. Activation of the p53 pathway was observed by Western blotting. Second, the same cell lines were used to generate innovative 3D-bioprinted models using 2% alginate and 5% gelatin bioinks. The 3D structures were treated with nutlin-3a for 72 h and assessed for viability using MTT or fixed and embedded in paraffin for histological and immunohistochemical investigation. Hematoxylin and eosin staining of non-treated 3D structures evidenced an architecture similar to the primary tumor rosette formation. Interestingly, nutlin-3a treatment significantly reduced the dimension of rosettes in both 3D models; additionally, it reduced the number of rosettes in the Y79 3D model. These data were supported by a significant reduction in proliferation and a decrease in Ki-67 expression. Our 3D models closely resemble retinoblastoma tumor tissue and can serve as a platform to assess innovative drugs or implement the promising results on the use of MDM2 inhibitors for retinoblastoma treatment.

## 1 Introduction

Retinoblastoma is the most prevalent retinal cancer affecting children under 5 years old and is characterized by uncontrolled proliferation (Alefeld et al., 2024). The outcome and survival rate significantly depend on the diagnosis and the rapidity of therapeutic interventions (Pritchard et al., 2016; Zhu et al., 2025). In high-income countries, retinoblastoma is considered a curable disease that, through early diagnosis and focused intervention, enables vision preservation in 90% of cases (Pritchard et al., 2016; Global Retinoblastoma Study Group, 2022). On the other hand, late diagnosis causes loss of vision in 50% of patients with advanced bilateral retinoblastoma and a 10-fold increase in mortality when extraocular metastasis is present (Pritchard et al., 2016; Global Retinoblastoma Study Group, 2022). The situation is more severe in children living in low-income countries, where delayed diagnosis and limited access to specialized care persist, resulting in poorer outcomes (Zhu et al., 2025).

Retinoblastoma cancer cells originate from post-mitotic cones with a mutation in the RB1 gene during development (Kaewkhaw and Rojanaporn, 2020). The consequent loss of Rb protein activates E2F family proteins, promoting uncontrolled proliferation (Engeland, 2022; Romani et al., 2022). In 95% of patients, a biallelic loss of RB1 is reported; however, tumor growth and progression are highly associated with additional genetic and epigenetic alterations, along with environmental interactions, which affect the disease course and drug treatment response (Kaewkhaw and Rojanaporn, 2020; Jo et al., 2020).

To date, early treatment options include photocoagulation, thermotherapy, cryotherapy, chemotherapy, and radiotherapy (Pritchard et al., 2016). In case of more-advanced tumors, the surgical removal of the eye (enucleation), in combination with chemotherapy, is performed to limit metastasis. Side effects of current treatments might impact the young patient’s quality of life with blindness and facial malformation, along with an increased risk of developing secondary tumors (Pritchard et al., 2016). In addition, resistance phenomena underscore the need to develop new low-toxic and highly specific therapeutic strategies.

Unlike many cancers, retinoblastoma is characterized by a p53 wild-type (p53^wt^) phenotype, making the pharmacological reactivation of the p53 pathway a realistic option to induce cell cycle block and apoptosis. Therefore, pharmacological targeting of the p53 pathway through MDM2 inhibitors is an attractive non-genotoxic approach for restoring tumor suppressor function (Romani et al., 2022; Elison et al., 2006).

Among MDM2 inhibitors, nutlins were the first class of active molecules synthesized to inhibit the MDM2–p53 interaction and prevent p53 degradation (Zhao et al., 2015; Wang et al., 2024). The active enantiomer nutlin-3a is known to stabilize p53 and activate its target genes, exerting a vast array of biological effects in preclinical models of p53^wt^ cancer cells of different origins, thereby restoring its biological activity (Romani et al., 2022; Wang and Yan, 2011; Psatha et al., 2023). Activated p53 induces a spectrum of downstream targets, including the expression of pro-apoptotic genes such as PUMA and BAX, which promote programmed cell death. Meanwhile, the induction of the p21 protein contributes to cell cycle arrest. Interestingly, p53 can also induce cell cycle arrest independently of p21 via TIGAR, a glycolytic regulator that redirects glucose flux toward the pentose phosphate pathway (PPP) (Bensaad et al., 2006).

To date, a reliable biomimetic model of retinoblastoma for drug testing is still lacking. For this reason, we created an innovative 3D-bioprinted model to better mimic tumor architecture and assess nutlin-3a efficacy as a feasibility study in a more complex model than the classical 2D approach. To this end, we used Y79 and Weri-Rb1 cell lines to build models of metastatic and non-metastatic tumors, respectively (Chévez-Barrios et al., 2000).

## 2 Materials and methods

### 2.1 2D cell cultures and treatments

Two immortalized retinoblastoma cell lines, Y79 and Weri-Rb1, both characterized by p53^wt^/Rb^mutated^, were used. Weri-Rb1 was selected as a model for non-metastatic retinoblastoma, whereas Y79 was selected as a model for invasive and metastatic tumors (Chévez-Barrios et al., 2000). Cell lines were acquired from the American Type Culture Collection (ATCC, Manassas, VA, United States) and maintained in RPMI 1640 (Carlo Erba, Milan, MI, IT) supplemented with 2 mM L-glutamine, 100 U/mL penicillin, and 100 mg/mL streptomycin (Sigma-Aldrich, St-Louis, MO, United States) and 20% or 10% fetal bovine serum (FBS; Gibco, Grand Island, NY, United States) for Y79 and Weri-Rb1, respectively. Cells were kept at 37 °C in a 5% CO_2_ and 90% relative humidity atmosphere. For the experimental assays, cells were seeded at a density of 5 × 10^5^ cells/mL and treated with nutlin-3a at concentrations ranging from 0.1 to 10 μM (0.1, 1, 2.5, 5, and 10 μM) or dimethyl sulfoxide (DMSO) (Sigma-Aldrich) as a control vehicle. Untreated cells were used as a negative control. Cells were passaged every 3 days and maintained at a concentration of 5 × 10^5^ cells/mL. *Mycoplasma* contamination was routinely monitored monthly using a MycoTest assay (InvivoGen, San Diego, CA, United States).

### 2.2 Cell viability, cell cycle profile, and apoptosis assays

The cytotoxic effects on the cells treated with nutlin-3a (0.1–10 μM) or DMSO were first evaluated using trypan blue (Sigma-Aldrich) dye exclusion counting after 24, 48, and 72 h.

The potential pro-apoptotic effect of nutlin-3a was evaluated via flow cytometry. After 24 and 48 h of treatment, cells were labeled with the annexin V-FITC/propidium iodide (PI) kit (Beckman Coulter Inc., Brea, CA, United States), following the manufacturer’s instructions. An FACSCalibur flow cytometer (BD Biosciences, San Jose, CA, United States) was used to collect data, and FlowJo™ v10.10 software (Tree Star, Ashland, OR, United States) was used for analysis.

At the same time points, the effects of nutlin-3a on the cell cycle profile of both cell lines were investigated using flow cytometry (Romani et al., 2024). In brief, after treatment, cells were incubated for 1.5 h at 37 °C with 5-bromodeoxyuridine (BrdU), which was incorporated into newly synthesized DNA. Afterward, cells were harvested and fixed with 70% ethanol and then kept at 4 °C until acquisition. All cells were subsequently stained with the anti-BrdU primary antibody (BD Pharmingen, San Diego, CA, United States) and the goat F(ab’)2 anti-mouse IgG (H + L) fluorescein isothiocyanate-conjugated secondary antibody (Beckman Coulter). Cells were stained with PI (50 μg/mL, Sigma-Aldrich) and acquired using a flow cytometer, FACSCalibur. The percentage of cells in each phase of the cell cycle was analyzed using FlowJo™ v10.10 software (Tree Star).

### 2.3 Western blotting

Twenty-four hours after treatment with nutlin-3a, cells were harvested for protein expression analysis by Western blotting, as previously described (Rimondi et al., 2021). Y79 and Weri-Rb1 cells were lysed in buffer containing 50 mM Tris-Cl, pH 7.5, 150 mM NaCl, 0.1% sodium dodecyl phosphate, 1% NP40 (NONIDET-P40), 0.25% mM sodium deoxycholate, and 1X Halt™ Protease and Phosphatase Inhibitor Cocktail (Thermo Scientific™, Waltham, MA, United States).

The bicinchoninic acid (BCA) Protein Assay Kit (Thermo Scientific™) was used to quantify the amount of protein in the lysates. Equal amounts of protein were separated on 10% or 12.5% SDS-PAGE gels and transferred onto nitrocellulose membranes (Cytiva, Marlborough, MA, United States). A specific molecular weight marker (Bio-Rad, Hercules, CA, United States) was used on each gel.

The following antibodies were used for immunoblotting: anti-p53 (clone DO-1), anti-MDM2 (clone SMP14), anti-PUMA (clone G-3), anti-TIGAR (clone G-2), and anti-E2F1 (clone KH95), all from Santa Cruz Biotechnology (Santa Cruz Biotechnology, Dallas, TX, United States), along with anti-p21 (polyclonal rabbit anti-human, Proteintech, Rosemont, IL, United States), anti-BAX (clone E4U1V, Cell Signaling, Danvers, MA, United States), and anti-tubulin (clone TUB 2.1, Sigma-Aldrich).

After incubation with secondary antibodies (polyclonal goat anti-mouse or anti-rabbit IgG peroxidase-conjugated; Sigma-Aldrich), specific band detection was performed using the enhanced chemiluminescent (ECL) lightning kit (Advansta Inc., San Jose, CA, United States). Images were acquired using the iBright™ CL1500 Imaging System, and band density was analyzed using iBright Analysis Software (Thermo Scientific™). Protein levels were normalized using tubulin protein expression as a loading control. For each experiment, the ratio versus the untreated value was then estimated. Statistical analyses were performed using the relative densities of a minimum of three separate experiments.

### 2.4 3D-bioprinted models

To prepare the hydrogel matrix, composed of 2% (w/v) alginate and 5% (w/v) gelatin, 0.2 g of alginate powder (Sigma-Aldrich) and 0.5 g of gelatin powder (Sigma-Aldrich) were sterilized under UV light for 30 min. The powders were then dissolved in 10 mL of sterile phosphate-buffered saline (PBS) (Sigma-Aldrich) at 60 °C under continuous stirring. The resulting hydrogel matrix was stored at 4 °C and used within a few days. Before use, the hydrogel matrix was placed at 37 °C for 30 min to obtain a liquid consistency suitable for cell incorporation.

Y79 and Weri-Rb1 cells were resuspended in the hydrogel matrix at final concentrations of 10 × 10^6^ cells/mL and 20 × 10^6^ cells/mL, respectively, and transferred to bioprinting cartridges. The printing cartridges were cooled at 4 °C for 10 min under continuous rotation to maintain homogeneous distribution of cells during matrix gelation. The cells suspended in the hydrogel matrix were printed at 20 °C using a 3D Allevi 3 3D bioprinter (Allevi, Philadelphia, PA, United States) to form a droplet/well in a 96-well plate. After printing, the droplets were cross-linked with 50 mM CaCl_2_ (Carlo Erba) for 5 min and rinsed with PBS as previously reported (Shi et al., 2017). 3D models were cultured in DMEM/F12 supplemented with 10% FBS, 2 mM L-glutamine, 100 U/mL penicillin, and 100 mg/mL streptomycin (Sigma-Aldrich). After 48 h, the bioprinted droplets were treated with 25 µM nutlin-3a, the equivalent concentration of DMSO (vehicle), or left in complete medium (untreated) for 72 h.

### 2.5 Paraffinization and hematoxylin and eosin staining of 3D-bioprinted models

The bioprinted drops were washed with PBS and afterward fixed in 10% buffered formalin (Sigma-Aldrich) saturated with 50 mM CaCl_2_ for 30 min. Bioprinted drops were left overnight in 70% ethanol and then washed with PBS. After dehydration in an ascending scale of alcohol and clarification in xylol (Diatech, Jesi, AN, IT), models were included in paraffin (Diatech) at a fusion temperature of 56–58 °C. Afterward, 5 µm tissue sections were cut with a microtome RM 2025 (Leica Biosystems, Nussloch, DE) and placed on glass slides. For the hematoxylin and eosin (H&E) staining, the sections were rehydrated using an escalating ethanol scale (100, 90, 80, and 70% 5 min each change) after being deparaffinized in two HistoClear (Histo-Line Laboratories, Pantigliate, MI, IT) passes, 10 min each. The hematoxylin solution (Histo-Line Laboratories) was used to stain the samples for 5 min. The samples were then rinsed twice in bi-distilled and tap water. After 20 s of staining with Eosin Y solution (Carlo Erba), the samples were incubated for 40 s each in 95% and 100% ethanol and for 1 min in HistoClear. DPX Mountant (Sigma-Aldrich) was then used to mount the slides. The images were acquired using an Aperio ScanScope® slide scanner with Aperio ImageScope v9.0.0.1516 software (Leica Biosystems). Quantification was determined using Fiji-ImageJ software. H&E staining was used to analyze the 3D-bioprinted models’ morphology. The ImageScope program was used to scan the slides, and a reference region was chosen at a ×20 magnification. Cell nuclei were then counted using Fiji software version 9.3.1 within the selected areas. The number of rosettes and their dimensions, expressed as area (mm^2^), were compared between controls and treated samples.

### 2.6 MTT assay

The viability of the 3D-bioprinted retinoblastoma models was assessed using the MTT assay. In brief, the bioprinted models, seeded and treated as previously described, were then incubated with the MTT (3-(4,5-dimethylthiazol-2-yl)-2,5-diphenyltetrazolium bromide) reagent (Roche Diagnostics Corporation, Indianapolis, IN, United States) for 1 h at 37 °C under standard culture conditions. Following incubation, a solubilization buffer was added directly to dissolve the formazan crystals and left overnight to ensure complete solubilization. The absorbance was measured at 570 nm using an Infinite M Plex microplate reader (TECAN, Männedorf, CH).

### 2.7 Ki-67 immunofluorescence

Tissue sections (5 μm) were deparaffinized in two xylene baths for 10 min each and subsequently rehydrated through a graded ethanol scale (100, 90, 80, and 60%), 5 min per step. After rehydration, sections were rinsed in distilled water, followed by PBS. Antigen retrieval was performed in citrate buffer (pH 6.0) using two microwave heating cycles: 5 min at maximum power followed by 5 min at medium power. Sections were allowed to cool for 30 min at room temperature and then washed three times with 1X Tris-buffered saline (TBS) for 10 min each. Blocking was assessed in 5% BSA prepared in 1X TBS containing 0.1% Tween-20 (Sigma-Aldrich). Sections were then incubated overnight in a humidified chamber with Ki-67 polyclonal antibody (27309-1-AP, Proteintech) at a 1:200 dilution in 0.5% BSA prepared in 1X TBS. After incubation, the slides were washed three times with 1X TBS (5 min each) and incubated with goat anti-rabbit Alexa Fluor 488 secondary antibody (A11008, Thermo Scientific™) at 1:100 in 0.5% BSA prepared in 1X TBS. Nuclear counterstaining was performed with DAPI. Finally, the sections were mounted using ProLong Gold Antifade Reagent (Thermo Scientific™) and imaged using fluorescence microscopy (Nikon Eclipse Ci equipped with a DS-Qi2 camera; Nikon, Shinagawa-ku, Tokyo, JP). Selected fields of identical dimension were cropped from the original images using Adobe Photoshop (Adobe, San Jose, CA, United States), without any adjustment to brightness or contrast.

### 2.8 Statistical analyses

Statistical analysis was performed using GraphPad Prism version 8 software (GraphPad Software, Boston, Massachusetts, United States). Normal distribution and homogeneity of variance of data obtained from at least three independent experiments were tested using the Shapiro–Wilk and Brown–Forsythe tests, respectively. The results were evaluated through analysis of variance (ANOVA), followed by Bonferroni’s *post hoc* test for multiple corrections. The results are expressed as the mean ± standard error of the mean (SEM), and significance was defined as *p-value* (*p*) < 0.05.

## 3 Results

### 3.1 Nutlin-3a reduces the viability of retinoblastoma through cell cycle block and apoptosis induction

**FIGURE 1:**
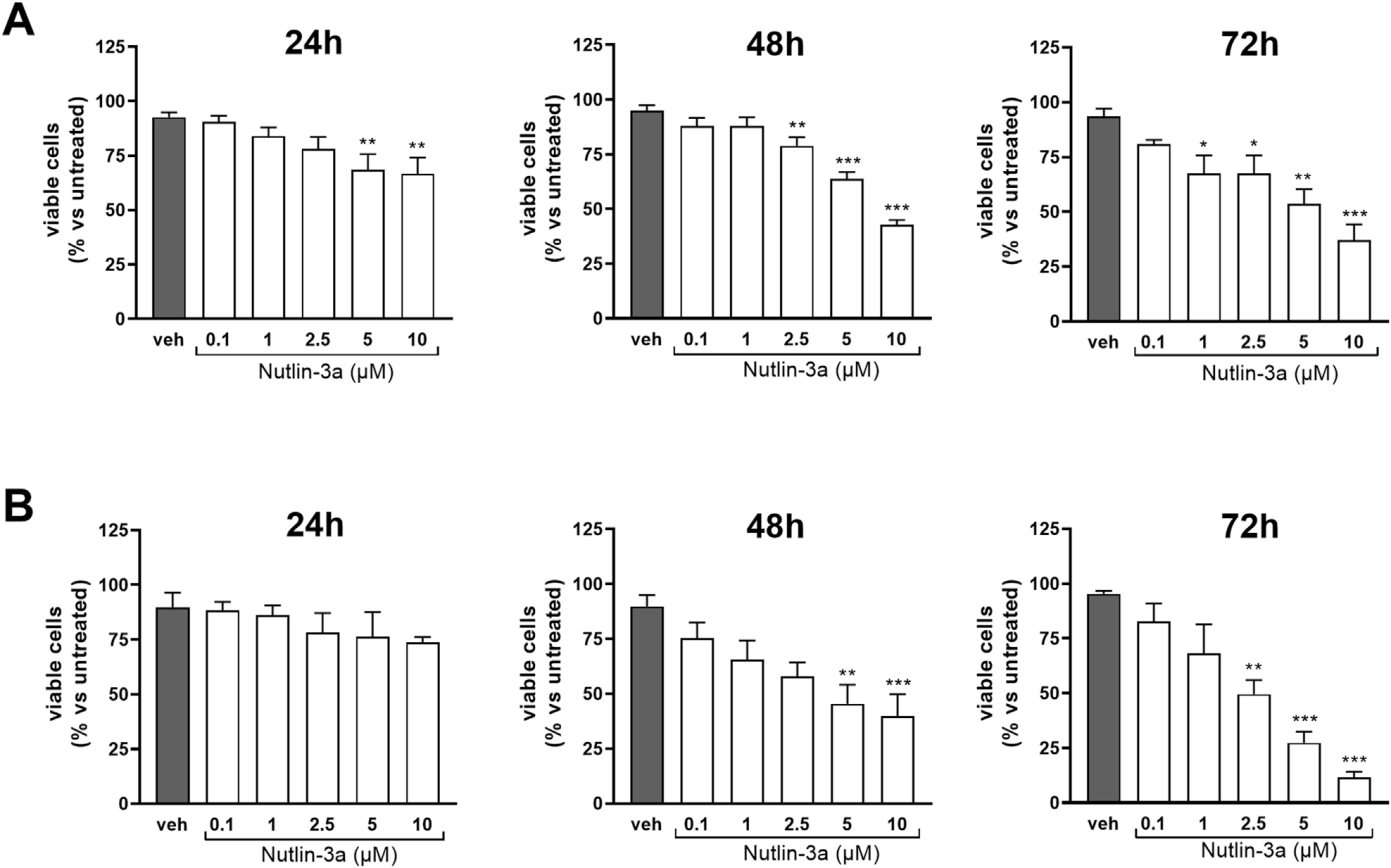
Nutlin-3a reduces the number of viable cells in Y79 and Weri-Rb1. The number of viable cells in Y79 **(A)** and Weri-Rb1 **(B)** was evaluated via trypan blue exclusion dye following 24, 48, and 72 h of treatment. Vehicle-treated cells (veh) are reported as controls. Bars represent the mean ± SEM from at least three independent experiments. Statistical analysis was performed via ANOVA followed by Bonferroni’s *post hoc* test. *p*-value (p): *, *p* ≤ 0.05; **, *p* ≤ 0.01; ***, *p* ≤ 0.001.

The biological effect of nutlin-3a was evaluated on Y79 and Weri-Rb1 cell lines as preclinical models of retinoblastoma. Cell viability was assessed 24, 48, and 72 h after treatment (Figure 1). Results showed a significant reduction in the number of viable cells in both retinoblastoma cell lines, with a significant reduction at the higher doses of nutlin-3a (5 and 10 µM), already evident at 24 h in Y79 cells (*p* < 0.01). A significant reduction in the number of viable cells was reached with longer treatment times. At 48 h, a significant reduction was reported starting from 2.5 µM (nutlin-3a, 2.5 µM, *p* < 0.01; 5 and 10 µM, *p* < 0.001). Additionally, after 72 h, nutlin-3a 1 µM also significantly reduced the number of viable cells (*p* < 0.05).

In Weri-Rb1, a significant reduction was reached only after 48 h (nutlin-3a, 5 µM, *p* < 0.01; 10 µM *p* < 0.001). The effect was enhanced after 72 h of treatment with a significant reduction also at 2.5 µM of treatment (nutlin-3a 2.5 µM, *p* < 0.01; 5 and 10 µM, *p* < 0.001).

The biological effects on apoptosis and cell cycle were assessed at 24 and 48 h.

**FIGURE 2:**
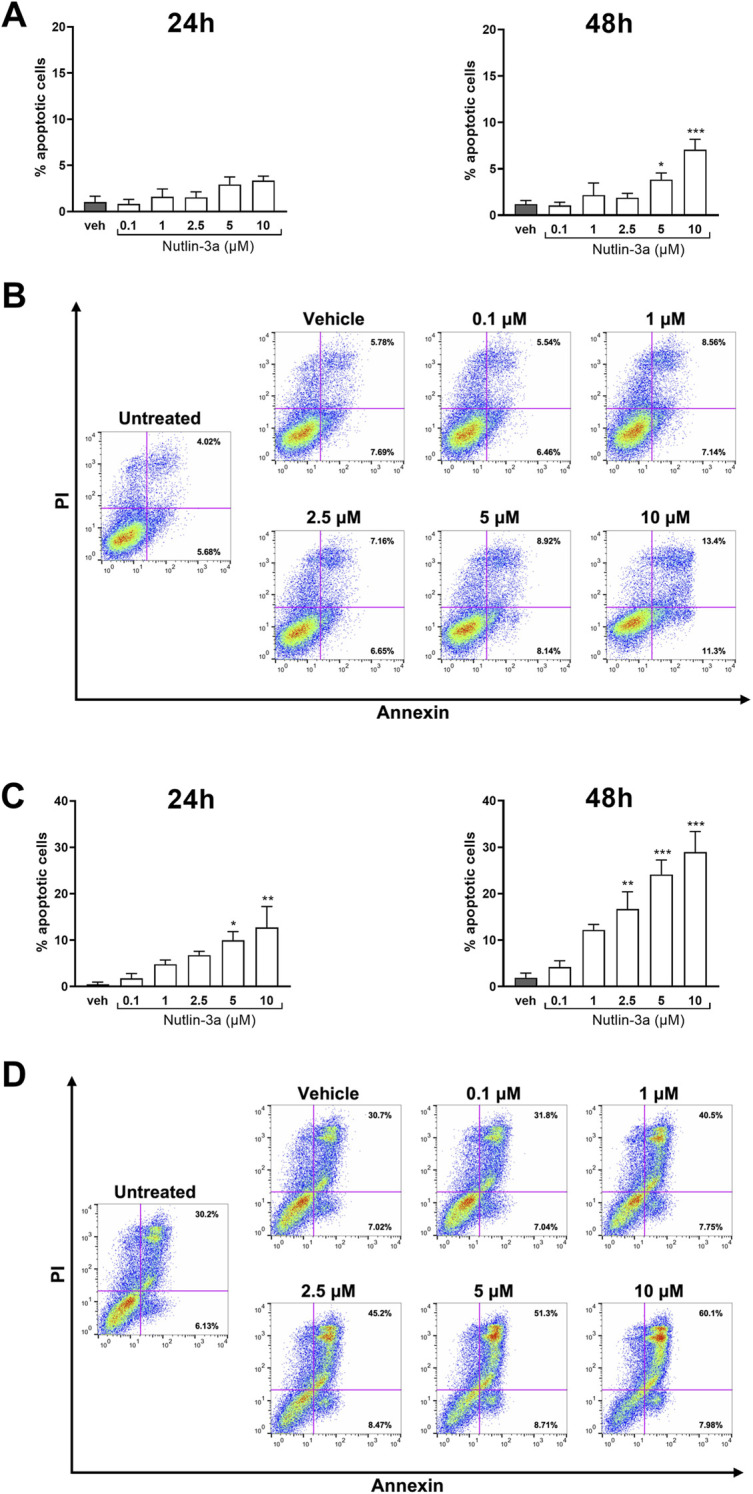
Nutlin-3a increases apoptosis in Y79 and Weri-Rb1. Apoptotic cells, at 24 and 48 h of treatment, in Y79 **(A)** and Weri-Rb1 **(C)**, are expressed as a percentage of the total cell population, normalized by subtracting the corresponding values of untreated controls (unt). Vehicle-treated cells (veh) are reported as controls. Bars represent the mean ± SEM from at least three independent experiments. Statistical analysis was performed via ANOVA followed by Bonferroni’s *post hoc* test. *p*-value (p): *, *p* ≤ 0.05; **, *p* ≤ 0.01; ***, *p* ≤ 0.001. In **(B,D),** representative dot-plots at 48 h of treatment are reported. The axis scales for fluorescence are reported as bi-exponential.

Treatment of Y79 cells with nutlin-3a showed a significant increase in the percentage of apoptotic cells at 48 h at concentrations of 5 and 10 μM (5 μM, *p* = 0.045; 10 μM, *p* < 0.001) (Figures 2A,B). Weri-Rb1 cells (Figures 2C,D) responded with a significant increase in apoptotic cells as early as 24 h (5 µM, *p* = 0.015; 10 µM, *p*-value<0.01), and the induction became significant also with 2.5 µM treatment for 48 h (2.5 µM, *p* < 0.01; 5 and 10 µM, *p* < 0.001).

**FIGURE 3:**
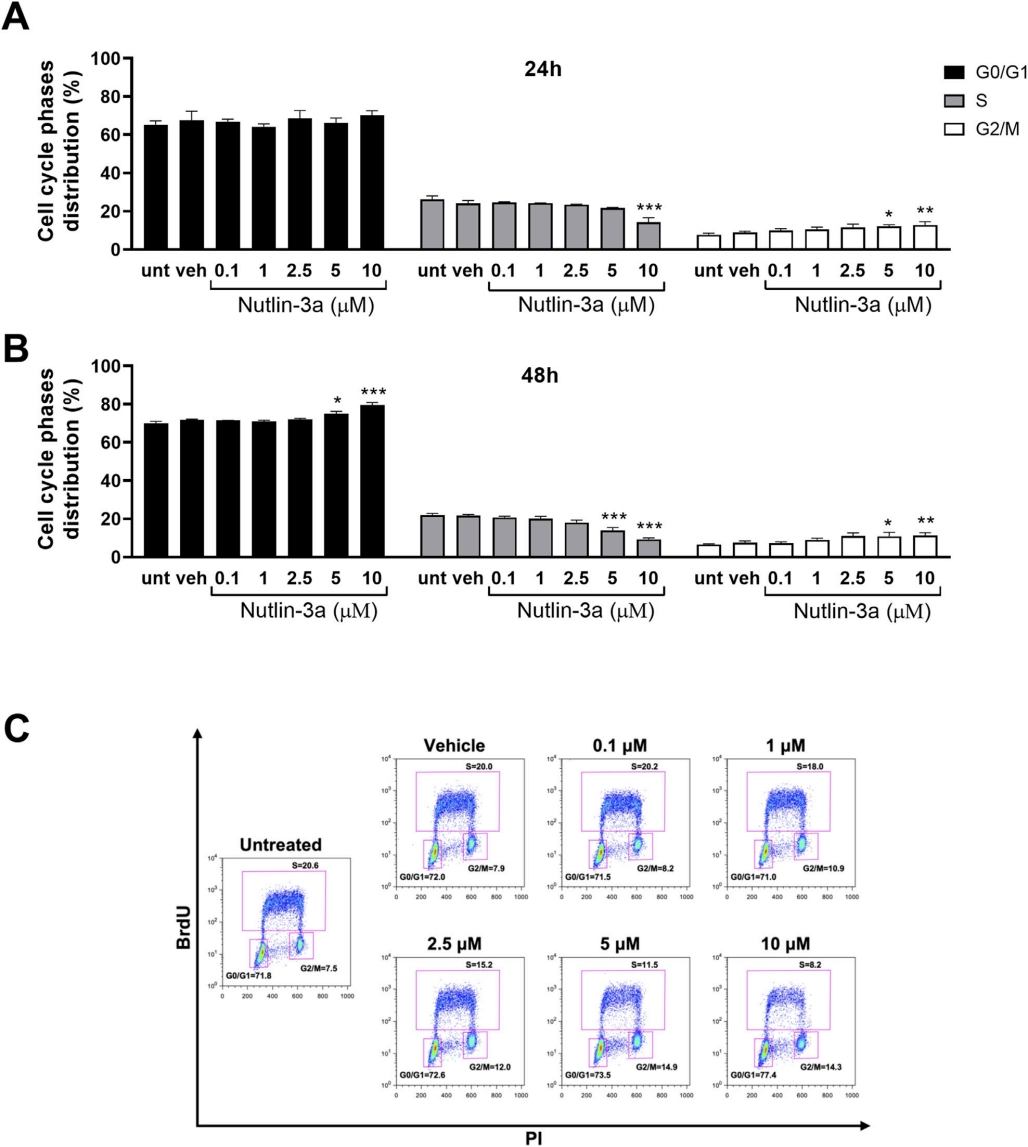
Nutlin-3a induces cell cycle block in Y79. The percentage of cells in each phase of the cell cycle is reported at 24 **(A)** and 48 h of treatment **(B)**. Untreated (unt) and vehicle-treated cells (veh) are reported as controls. Bars represent the mean ± SEM from at least three independent experiments. **(C)** Representative dot-plot at 48 h of treatment. The BrdU axis scale is reported as logarithmic, the PI axis scale is reported as linear. Statistical analysis was performed via ANOVA followed by Bonferroni’s *post hoc* test. *p*-value (p): *, *p* ≤ 0.05; **, *p* ≤ 0.01; ***, *p* ≤ 0.001.

**FIGURE 4:**
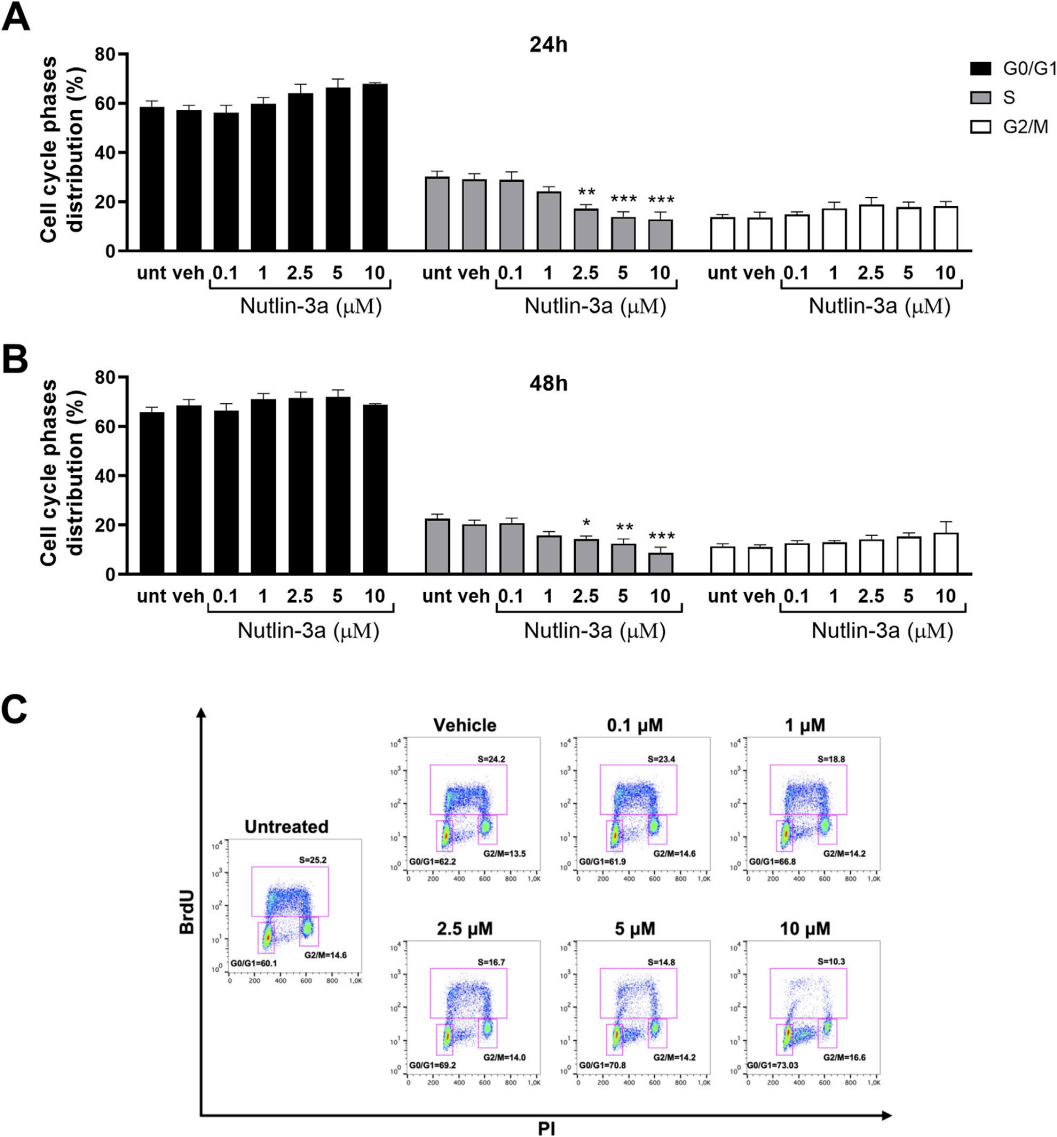
Nutlin-3a induces cell cycle block in Weri-Rb1. The percentage of cells in each phase of the cell cycle is reported at 24 **(A)** and 48 h of treatment **(B)**. Untreated (unt) and vehicle-treated cells (veh) are reported as controls. Bars represent the mean ± SEM from at least three independent experiments. **(C)** Representative dot plot at 48 h of treatment. Statistical analysis was performed via ANOVA followed by Bonferroni’s *post hoc* test. *p*-value (p): *, *p* ≤ 0.05; **, *p* ≤ 0.01; ***, *p* ≤ 0.001.

As shown in Figures 3, 4, the data indicate cytostatic effects on retinoblastoma cell lines. In Y79 cells (Figure 3), only the higher concentration of nutlin-3a, after 24 h of treatment, induced a significant reduction in the percentage of cells in the S-phase (10 µM, *p* < 0.001) and a consequent increase in the percentage of cells in the G2/M phase (5 µM, *p* = 0.043; 10 µM, *p* < 0.01). After 48 h of treatment, cell cycle block on S and G2/M phases became significant both with nutlin-3a treatment 5 and 10 µM (S-phase, *p* < 0.001; G2/M phase: 5 µM, *p* = 0.044; 10 µM, *p* < 0.01), with an additional increase in the percentage of cells in the G0/G1 phase (5 µM, *p* = 0.015; 10 µM, *p* < 0.001).

In Weri-Rb1 cells (Figure 4), nutlin-3a induced a significant cell cycle block, with a significant reduction in the percentage of cells in the S-phase at 2.5, 5, and 10 µM, both after 24 h (2.5 µM, *p* < 0.01; 5 and 10 µM, *p* < 0.001) and 48 h of treatment (2.5 µM, *p* < 0.02; 5 µM, *p* = 0.0048 and 10 µM, *p* < 0.001).

### 3.2 Nutlin-3a-mediated cytotoxicity depends on p53 pathway activation in retinoblastoma

**FIGURE 5:**
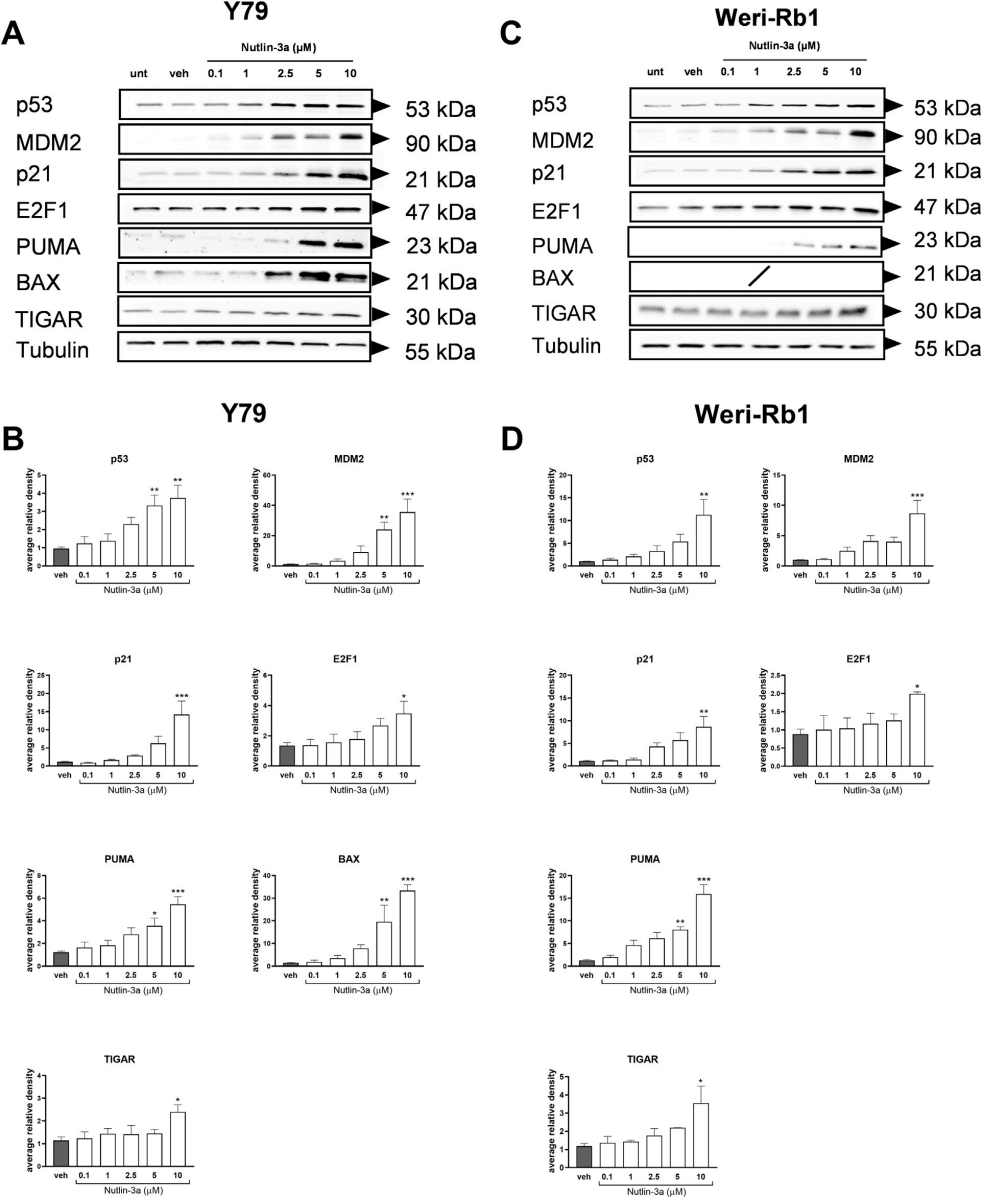
Nutlin-3a increases the expression of p53 pathway-related proteins including pro-apoptotic targets (BAX and PUMA) and the metabolic regulator TIGAR. Nutlin-3a induces a dose-dependent upregulation of p53 pathway-related proteins, including the pro-apoptotic markers BAX and PUMA, and the metabolic regulator TIGAR, following 24 h of treatment. Representative Western blots from three independent experiments are shown in **(A)** for Y79 and **(C)** for Weri-Rb1. Protein levels were quantified using densitometric analysis, normalized to tubulin (loading control), and expressed in arbitrary units for Y79 **(B)** and Weri-Rb1 **(D)**. Vehicle-treated cells (veh) are reported as controls. Data are presented as the mean ± SEM from three independent biological replicates. Statistical analysis was performed via ANOVA followed by Bonferroni’s *post hoc* test. *p*-value (p): *, *p* ≤ 0.05; **, *p* ≤ 0.01; ***, *p* ≤ 0.001.

To investigate the action of nutlin-3a on retinoblastoma cells at the molecular level, we analyzed the p53 pathway. Twenty-four hours of treatment resulted in a significant upregulation of the p53 protein and its target protein, MDM2, in both cell lines. In Y79 cells, significant increases in protein levels were reported at both 5 and 10 µM of treatment (p53: *p* < 0.01 at 5 and 10 μM; MDM2: *p* < 0.01 and *p* < 0.001 at 5 and 10 μM, respectively), as shown in Figures 5A,B. In Weri-Rb1, only the highest concentration of nutlin-3a induced a significant upregulation of p53 (*p* < 0.01) and MDM2 (*p* < 0.001) proteins, both shown in Figures 5C,D. The cytostatic effect of nutlin-3a was confirmed by significant upregulation of p21 (Y79: *p* < 0.001; Weri-Rb1: *p* < 0.01) and E2F1 proteins (Y79: *p* = 0.049; Weri-Rb1: *p* = 0.039) with 10 µM treatment. The highest treatment concentration significantly increased TIGAR protein levels in both cell lines (Y79: *p* = 0.030; Weri-Rb1: *p* = 0.011), suggesting its possible contribution to p53-mediated cell cycle arrest (Tang et al., 2021). We also investigated relevant p53-dependent, pro-apoptotic proteins. In both cell lines, the significant upregulation of the PUMA protein was reported as a pro-apoptotic driver, significantly upregulated by nutlin-3a treatment at 5 and 10 µM (Y79: *p* = 0.023 and *p* < 0.001; Weri-Rb1: *p* < 0.01 and *p* < 0.001). Moreover, in Y79 cells only, we reported a significant upregulation of the BAX protein (*p* < 0.01 and *p* < 0.001 at 5 and 10 μM, respectively), which supports the involvement of the mitochondrial apoptotic pathway. On the other hand, in Weri-Rb1, which is known not to express the BAX protein at basal levels, nutlin-3a was unable to induce its transcription.

### 3.3 Nutlin-3a influences the morphology of 3D-bioprinted models

**FIGURE 6:**
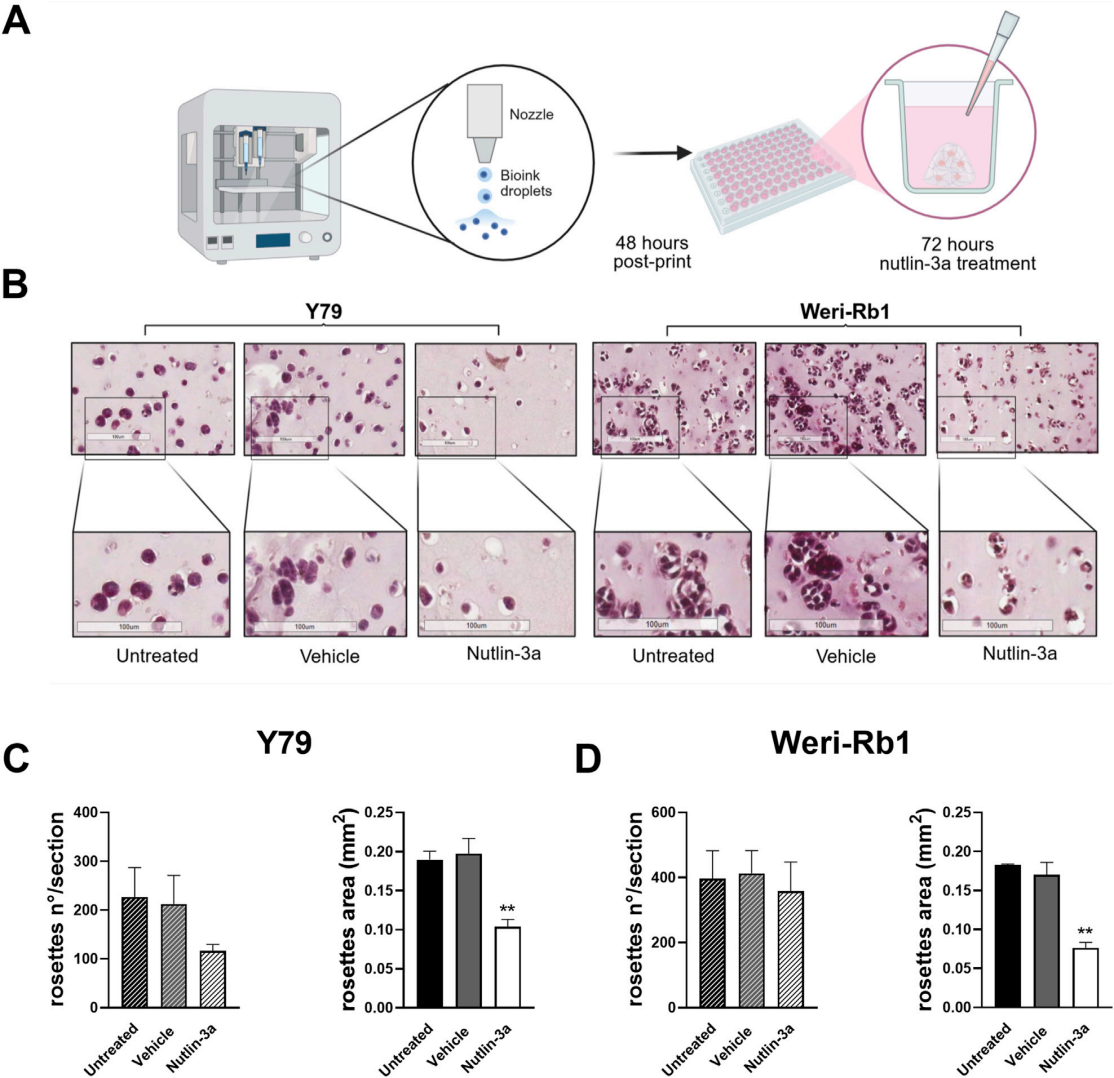
Schematic representation of the bioprinted 3D model. The protocol and treatment conditions are reported in **(A)**. Created in Biorender: https://BioRender.com/mex8t1o. Representative H&E-stained images illustrate morphological differences between untreated, vehicle-treated, and nutlin-3a-treated bioprinted models. Samples treated with nutlin-3a exhibit a significant decrease in both cell number and size, compared to the untreated and vehicle groups **(B)**. Quantification of cell numbers and rosette areas in response to nutlin-3a treatment is shown for Y79 **(C)** and Weri-Rb1 **(D)** 3D models. Statistical analysis was performed via ANOVA followed by Bonferroni’s *post hoc* test. *p*-value (*p*): **, *p* ≤ 0.01.

To better mimic the complex *in vivo* tumor, we generated a 3D-bioprinted model using an alginate–gelatin matrix to evaluate the morphology of tumor cells and their response to nutlin-3a with respect to 2D cultures (Figure 6A). Nutlin-3a concentration and time of treatment in 3D models were increased compared to the 2D culture, based on model complexity. The bioprinted models treated with 25 µM nutlin-3a for 72 h, fixed in PFA, and included in paraffin were histochemically assessed using H&E. As reported in Figure 6B, in the 3D model, both retinoblastoma cells formed a cellular aggregate that recapitulates an *in vivo* tumoral rosette. Nutlin-3a treatment significantly reduced the size of these structures in Y79 cells (*p* = 0.0023 vs. untreated), and a trend toward a reduction in the number of rosettes was appreciable (Figure 6C). Similarly, in Weri-Rb1 cell models, data suggested a significant variation in the architecture of the structures when treated with nutlin-3a (*p* = 0.0012 vs. untreated), without changing the number of rosettes (Figure 6D). The absence of significant variation in rosette-like structures in both cell lines is consistent with the timing of treatment. Three-dimensional (3D) models were allowed to grow for 48 h before treatment. This timeframe enabled the cells to recover from bioprinting stress and organize into the matrix, forming rosette-like nuclei of growth. After this initial recovery phase, treatment with nutlin-3a, inhibited rosette expansion by inducing cell cycle arrest and apoptosis.

### 3.4 Nutlin-3a reduces the proliferation of 3D-bioprinted models

**FIGURE 7:**
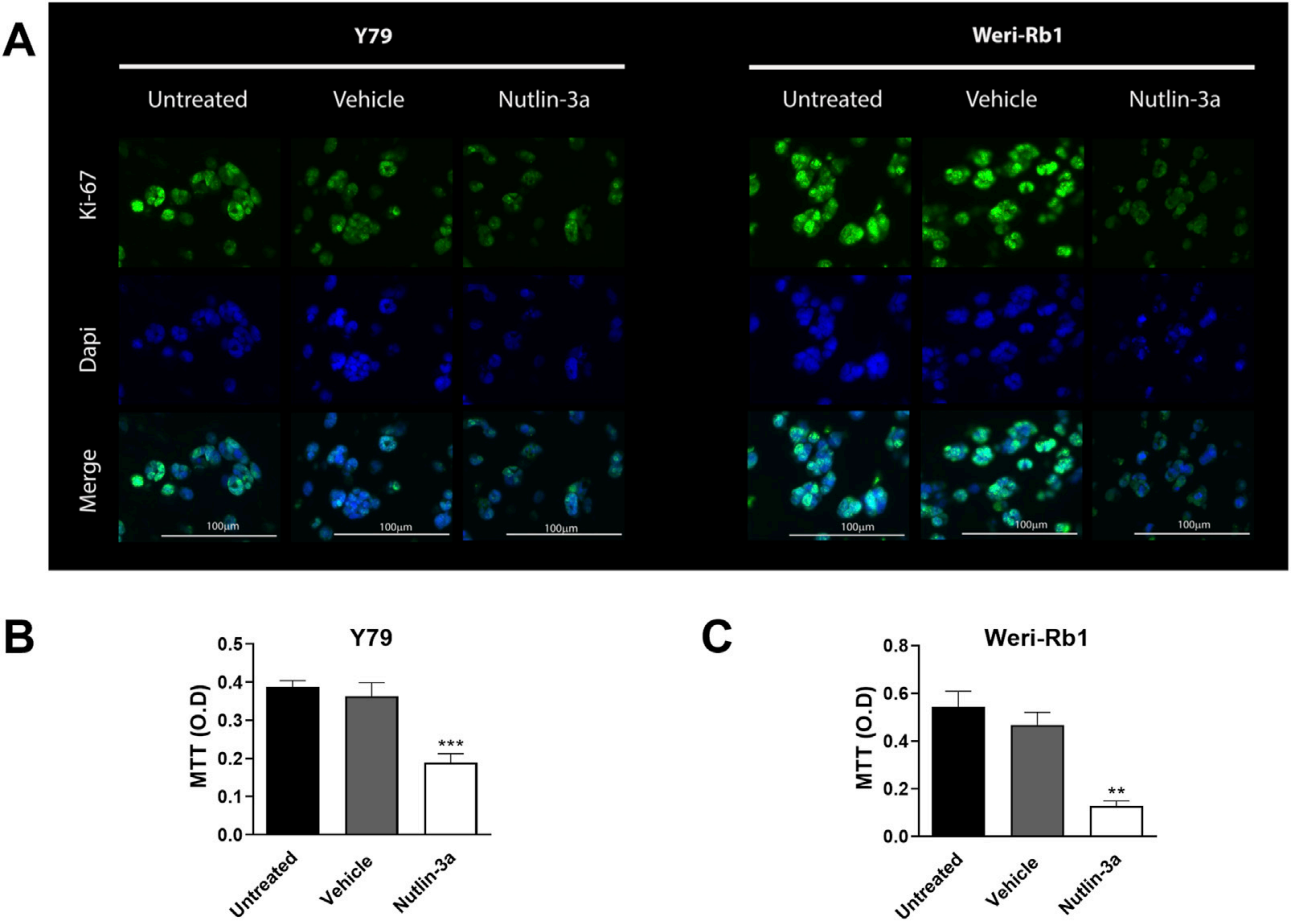
Nutlin-3a reduces proliferation in Y79 and Weri-Rb1 in the 3D bioprinted model. Ki-67 expression in both 3D models is shown in **(A)** following 72 h of treatment; Ki-67 immunofluorescence staining is reported in green; nuclei are stained with DAPI in blue, and merged images were reported. Magnification ×20; scale bar 100 µm. MTT reduction in Y79 **(B)** and Weri-Rb1 **(C)** is reported as optical density (O.D.). Untreated (unt) and vehicle-treated (veh) cells are reported as controls. Bars represent the mean ± SEM from at least three independent experiments. Statistical analysis was performed via ANOVA followed by Bonferroni’s *post hoc* test. *p*-value (*p*): **, *p* ≤ 0.01; ***, *p* ≤ 0.001.

Bioprinted models were also used to assess the influence of nutlin-3a on cell proliferation. In both retinoblastoma cell lines, after 3 days of treatment, data showed a significant reduction in cell proliferation, as observed using Ki-67 staining (Figure 7A). Ki-67 immunofluorescence staining evidenced a distinct nuclear localization of the protein, which was reduced in treated samples. In agreement, our data showed a significant decrease in MTT reduction compared to untreated samples both in Y79 (*p* < 0.001, Figure 7B) and Weri-Rb1 (*p* = 0.0023, Figure 7C). These data suggest a cumulative effect based on a reduction in the number of cells and their metabolic activity.

## 4 Discussion

Retinoblastoma still causes several deaths in low–middle-income countries, where 80% of worldwide cases occur (Ramanathan and Vijayasekharan, 2023). In contrast, in high-income countries, early diagnosis can preserve vision and eyes, but more frequently, resistance phenomena increase the chance for recurrence or the development of secondary tumors (Pritchard et al., 2016).

The present study investigates the use of nutlin-3a as a proof of concept for the use of MDM2 inhibitors as non-genotoxic approaches for retinoblastoma. Ellison et al. (2006) previously suggested nutlin-3a as an efficient treatment to induce p53-dependent apoptosis and p21-dependent cell cycle arrest in *in vitro* retinoblastoma cell lines. In agreement, our study confirms the efficient activation of the p53 pathway, with the involvement of PUMA in inducing apoptosis and p21-mediated cell cycle arrest, in both retinoblastoma cell lines tested. These results suggest drug efficacy in metastatic and non-metastatic tumors, as represented by the Y79 and Weri-Rb1 cell lines, respectively (Chévez-Barrios et al., 2000). Activation of the intrinsic apoptotic pathway, driven by p53, was confirmed by the increased levels of pivotal proteins involved, such as PUMA and BAX, in Y79 cells. These data support the involvement of the canonical pathways with mitochondrial dysfunction, mediated by BAX, in association with cytochrome c and caspases in the cell death program.

Nevertheless, in Weri-Rb1 cells, although p53 and PUMA are upregulated, nutlin-3a does not induce BAX protein expression. This cell line was previously reported not to express the BAX protein; although at low levels, certain treatments, such as topotecan or cationic antimicrobial dodecapeptides, can induce its expression (Suresh Babu et al., 2022). Considering that the biological data confirmed a significant induction of apoptosis in Weri-Rb1 cells, we suggest that cell death induced by nutlin-3a is driven by PUMA, which we hypothesize acts on other pro-apoptotic proteins, such as BAD or BAK, instead of BAX, to induce mitochondrial depolarization. This pathway is already reported in other cancer cells (prostate cancer and colon carcinoma) to induce apoptosis to overcome the absence of BAX (Hemmati et al., 2006). This promising biological activity in controlling retinoblastoma cells was observed in 2D *in vitro* culture, which does not fully replicate in *in vivo* tumor histology. To date, a reliable biomimetic model for drug testing or a representative animal model for retinoblastoma is still lacking. Organoid models proposed in the literature are a valuable replacement for animal studies; however, they focus on the differentiation of patient-derived stem cells, which might increase the difficulties in data interpretation derived from basic drug testing due to patients’ variability (Liu et al., 2021; Norrie et al., 2021). Based on this, we generated bioprinted 3D models, including the same cell lines tested in 2D, to verify the data obtained in a structure that better represents tumor architecture. We characterized the biomimetic properties of the 3D model (architecture and viability) and assessed the nutlin-3a treatment response. Our models generate complex viable structures that recapitulate the rosette-like formation of an *in vivo* tumor (Das et al., 2014). Treatment of these models with nutlin-3a confirmed the drug’s efficacy in reducing tumor growth.

In our study, nutlin-3a was applied at concentrations up to 10 µM in traditional 2D monolayer cultures, while in the 3D model, a higher concentration of 25 µM was required to achieve a comparable reduction in cell viability. This difference reflects not only the increased structural complexity of 3D systems but also the physiological barriers that reduce drug availability within the tumor-like microenvironment. As highlighted by Cavaco et al. (2021) in their work on cancer spheroids, 3D culture systems often exhibit lower drug sensitivity than 2D cultures, requiring higher concentrations of therapeutic agents to achieve comparable cytotoxic effects. Their findings showed that cancer spheroids display higher IC_50_ values due to limited drug penetration, gradients in oxygen, and nutrient distribution (Cavaco et al., 2021). These mechanisms are also relevant in our retinoblastoma bioprinted models. Consistent with this knowledge, nutlin-3a often requires higher concentrations in 3D models to exert its therapeutic effect, likely due to limited drug diffusion into the tumor core. Nevertheless, at 25 μM, nutlin-3a demonstrated functional efficacy in our 3D retinoblastoma model. The effect was evidenced by a reduction in rosette-like structures and a decrease in Ki-67 expression. At the same time, based on cell nuclei staining analysis on histological sections, we can hypothesize a reduced apoptosis rate in the 3D model compared to the 2D counterpart. These findings suggest that, despite the architectural complexity, nutlin-3a retains its biological activity when administered at sufficient doses.

This makes our model an innovative platform for testing new potential pharmacological approaches. Three-dimensional bioprinting generates standardized models, which improve the reproducibility of the data obtained. Although three-dimensional models of retinoblastoma have already been developed, some of which have demonstrated the presence of rosette-like structures (Liu et al., 2020), our model stands out due to the use of a highly reproducible bioprinted platform and the incorporation of an innovative treatment: nutlin-3a. To date, no studies have specifically addressed the functional response of rosettes to targeted therapies in 3D models. In this context, our work represents an original contribution as it introduces the possibility of assessing pharmacological efficacy in relation to distinct histopathological structures typical of retinoblastoma, potentially opening new avenues for more targeted and effective therapeutic strategies.

A key strength of this bioprinted model is its modularity, allowing the integration of additional cell types physiologically present in the tumor microenvironment. A future improvement for reaching a more physiological relevance can be the incorporation of retinal epithelial cells, such as ARPE-19, to mimic the epithelial ocular barrier and maintain tissue homeostasis (Esposito et al., 2024). Additionally, the inclusion of retinal endothelial cells, such as ACBRI 181, modeling the blood–retinal barrier and tumor–vasculature interactions in ocular contexts, would support the reconstruction of a vascular-like network. Additionally, astrocytes can also be introduced to better replicate the neural microenvironment, given their role in maintaining central nervous system homeostasis and mediating interactions with tumor cells (Yoo et al., 2021). The integration of epithelial, endothelial, and glial cells in 3D models will allow the development of advanced physiologically relevant tumor models for more accurate preclinical evaluation of therapeutic strategies. Although achieving this complexity can be challenging, the monoculture models we demonstrated, sensitive to MDM2 inhibition, serve as a starting point for designing and bioprinting a more integrated model.

Although it has promising anticancer properties, nutlin-3a is limited by poor pharmacokinetics, which restricts its clinical application. To overcome these limitations, other new-generation MDM2 inhibitors were developed and already assayed in clinical trials for solid and hematologic cancers. In addition, recent advances in nanoparticle-based delivery systems, such as ethosomes and transethosomes, have demonstrated the potential for use as an ophthalmic formulation for local treatment. These strategies may enhance intratumoral drug distribution and improve therapeutic outcomes in the 3D model (Romani et al., 2024). In this context, Brennan et al. (2011) demonstrated that localized delivery via the subconjunctival route can effectively bypass the blood–ocular barrier in a preclinical model. This approach allows for high intraocular drug levels while minimizing systemic exposure and associated toxicity (Brennan et al., 2011). Based on these findings, therapeutic strategies for nutlin-3a in the eye may be achievable through targeted delivery methods that concentrate the drug within ocular compartments, such as using nanodelivery systems, including liposomes. These lipid-based nanocarriers not only enhance the stability and bioavailability of nutlin-3a but also facilitate targeted delivery and controlled release to preserve molecular stability and thereby maintain efficacy without compromising systemic safety (Pant et al., 2012). Notably, nutlin-3a-loaded ethosomes have shown enhanced anti-melanoma activity through p53-mediated apoptosis (in HT144 cells), highlighting their therapeutic potential in skin cancers (Romani et al., 2024). Moreover, similar formulations for nutlin-3a delivery have been effective in preventing UV-induced skin damage, suggesting broader dermatological applications (Esposito et al., 2024). Importantly, these nanosystems can be formulated for topical or ocular administration (Brennan et al., 2011; Romani et al., 2022), including as eye drops, which is particularly advantageous in resource-limited settings where conventional systemic therapy may be less accessible. Therefore, incorporating nutlin-3a into nanoparticulate carriers represents a promising strategy to improve its therapeutic index and accessibility. Further studies are warranted to optimize these formulations and evaluate their clinical efficacy *in vivo*.

## Data Availability

The raw data supporting the conclusions of this article will be made available by the authors, without undue reservation.
